# Quantifying Voice Characteristics for Detecting Autism

**DOI:** 10.3389/fpsyg.2021.665096

**Published:** 2021-09-07

**Authors:** Meysam Asgari, Liu Chen, Eric Fombonne

**Affiliations:** ^1^Institute on Development and Disability, Department of Pediatrics, Oregon Health & Science University, Portland, OR, United States; ^2^Departments of Psychiatry and Pediatrics, Oregon Health & Science University, Portland, OR, United States

**Keywords:** voice, speech analysis, autism spectrum disorder, harmonic model, prosody, machine learning

## Abstract

The presence of prosodic anomalies in autistic is recognized by experienced clinicians but their quantitative analysis is a cumbersome task beyond the scope of typical pen and pencil assessment. This paper proposes an automatic approach allowing to tease apart various aspects of prosodic abnormalities and to translate them into fine-grained, automated, and quantifiable measurements. Using a harmonic model (HM) of voiced signal, we isolated the harmonic content of speech and computed a set of quantities related to harmonic content. Employing these measures, along with standard speech measures such as loudness, we successfully trained machine learning models for distinguishing individuals with autism from those with typical development (TD). We evaluated our models empirically on a task of detecting autism on a sample of 118 youth (90 diagnosed with autism and 28 controls; mean age: 10.9 years) and demonstrated that these models perform significantly better than a chance model. Voice and speech analyses could be incorporated as novel outcome measures for treatment research and used for early detection of autism in preverbal infants or toddlers at risk of autism.

## Introduction

Autism spectrum disorder (ASD) comprises a range of developmental impairments affecting social communication and patterns of play and behaviors (American Psychiatric Association, 2013). Symptoms emerge in early life and often lead to long-lasting impairments over the life span (Mubashir et al., 2020). Although language delays and structural language deficits are frequently observed in the development of individuals with autism, language delays are not necessary diagnostic criteria. However, pragmatic impairments of verbal and non-verbal communications are a key feature of autism at different developmental stages. About 10% of school-age children are non-verbal and an additional 20% are minimally verbal. Lack of language development in childhood predicts long term negative outcomes in adult life. Reflecting the critical importance of language, early intensive behavioral interventions target communication skills, whether verbal or non-verbal, to improve developmental trajectories of young children with autism.

In addition to the language deficits, prior studies have well established the impact of ASD on prosodic aspects of speech (Sheinkopf et al., 2000; Shriberg et al., 2001). Prosody refers to the set of speech variables including rhythm, loudness, stress, rate of speech, pitch, and intonation that modulate human communications (Waibel, 1988). For example, emotional state of the speaker is conveyed through prosodic elements of speech. Also, the form of a sentence, such as declarative (statement) and interrogative (question) sentences, are often expressed through varying pitch and intonation. Additionally, acoustic and prosodic properties may provide considerable insight into human health (Asgari and Shafran, 2018). In children with ASD, atypical patterns in prosodic elements such as monotonous pitch (Sharda et al., 2010), reduced stress (Shriberg et al., 2001), odd rhythm (Trevarthen and Daniel, 2005), flat intonation (Cooper and Hanstock, 2009), and even differences in harmonic structure of their speech (Bonneh et al., 2011) are among the earliest signs of the disorder. Prior research has shown a strong relationship between prosodic abnormalities in individuals with ASD and their social and communicative abilities (Shriberg et al., 2001; Paul et al., 2005; Fusaroli et al., 2017). In addition, prosodic abnormalities have been shown to be familial and to index genetic liability to ASD (Patel et al., 2020). Recent reviews of prosody studies in ASD can be found in the published literature (Loveall et al., 2021; Zhang et al., 2021).

These findings highlight the importance of fine-grained assessment of prosodic elements for detecting, diagnosing, and monitoring of ASD. Despite a continued interest in characterizing acoustic and prosodic abnormalities in ASD to potentially exploit them in objective evaluations, their measurement in clinical settings has been notoriously difficult. To give an example, only one item of the Autism Diagnostic Observation Schedule (ADOS-2; Lord et al., 2003) Module 3 (item A2) specifically rates “Speech abnormalities associated with autism (Intonation/Volume/Rhythm/Rate)” on a 0–2 scale where an abnormal score of 2 represents a composite of abnormal features crudely lumped together. Likewise, only one item from the Social Responsiveness Scale (SRS; Constantino and Gruber, 2005) evaluates a limited aspect of voice quality [item 53: talks to people with an unusual tone of voice (for example, talks like a robot or like he/she is giving a lecture)]. Furthermore, there is no reference to speech or voice quality in any of the 7 diagnostic criteria laid out in the fifth version of Diagnostic and Statistical manual of Mental disorders (DSM-5; American Psychiatric Association, 2013). To improve on observational ratings, use of voice recording and voice analysis is therefore necessary. Recently, speech researchers have proposed automated methods for assessment of prosody (van Santen et al., 2009; Hönig et al., 2010; Truong et al., 2018; Truong et al., 2018). Despite their potential benefits, a major challenge in these systems is the lack of computational algorithms that could extract robust and accurate prosodic measures, such as pitch. There are several pitch detection algorithms (PDAs) in the literature (Talkin et al., 1995; Boersma and Weenink, 2001; de Cheveigné and Kawahara, 2002; Sun, 2002; Kawahara et al., 2008; Drugman and Alwan, 2011) that produce accurate results for highly periodic signals recorded in noise-free environments. Yet, due to the physical structure of the vocal tract and to the noise factor observed in disordered speech, the speech signal is not perfectly periodic and often described as quasi-periodic signal. Thus, false detection [“octave errors” (Kumaraswamy and Poonacha, 2019)] often occur that result in estimating the pitch by half or double the true value. Another limitation of existing PDAs for analyzing pathological speech is the lack of sufficient time-frequency resolution for capturing fine perturbation of pitch and amplitude of voiced speech –a common phenomenon of disordered speech. One notable exception for estimating key prosodic measures is the harmonic model (HM) of speech described in the next section. However, the straight forward application of this model leads to certain drawbacks such as “octave errors” in pitch estimation problem (Asgari and Shafran, 2013).

In our prior work, we mitigated these drawbacks by modifying the HM and introduced an improved version of HM, known as time-varying harmonic model (TV-HM) of speech, that achieves more accurate and reliable estimation of acoustic and prosodic measures of speech (Asgari and Shafran, 2018). We successfully adapted TV-HM for characterizing speech impairments in clinical populations including Parkinson’s disease (Asgari and Shafran, 2010a, b) and clinical depression (Asgari et al., 2014). In this study, we extended these methodological improvements of the TV-HM to youth with autism in order to better characterize the atypical patterns of prosodic properties in their speech. By comparing speech samples collected with standardized ADOS-2 (Lord et al., 2003) procedures in youth with or without ASD, our study objectives were to: (1) examine if analysis of voice and speech quality only could predict diagnostic membership better than the chance model; and (2) test if speech samples collected in specific ADOS-2 tasks were or not equivalent in differentiating the 2 groups of children and adolescents, and if the combination of speech samples across tasks was improving performance over single tasks samples.

## Materials and Methods

### Participants

Participants with either ASD or typical development (TD) were recruited by community outreach and referrals from OHSU specialized clinics to participate in neuroimaging study. All participants came in for a screening visit to determine if they qualified for the study. Informed written consent or assent was obtained from all participants and their parents who also had to be fluent in English. All youth in the ASD group had their diagnosis confirmed (using DSM-5 criteria) by a research diagnostic team that included an experienced child psychiatrist and a clinical psychologist, after review of standardized diagnostic assessments (both videos and scored protocols) and using best-estimate procedures. ASD was ruled out in TD youth based on ADOS-2 and SRS scores supplemented by expert clinical review. Exclusion criteria for all groups included the presence of seizure disorder, cerebral palsy, pediatric stroke, history of chemotherapy, sensorimotor handicaps, closed head injury, thyroid disorder, schizophrenia, bipolar disorder, current major depressive episode, fetal alcohol syndrome, Tourette’s disorder, severe vision impairments, Rett’s syndrome, currently taking psychoactive medications, and an IQ below 70. A total of 132 subjects with ASD and TD were recruited for the neuroimaging study. Of the 104 participants with ASD, 14 participants were excluded due to poor recording quality, leaving a total sample size of 118 for analysis (90 ASD, 28 TD).

### Diagnostic, Cognitive and Behavioral Assessments

#### Autism Diagnosis Observation Schedule

The ADOS-2 (Lord et al., 2003) is a semi-structured, standardized assessment in which a trained examiner engages participants in activities that are designed to elicit social and communication behaviors indicative of symptoms of ASD as defined in the DSM-5. In this study, all participants were administered the Module 3 of the ADOS-2 that is suitable for children and adolescents with fluent speech. Module 3 comprises 14 tasks that are generally administered in sequence although the tester has some flexibility to change the task order if clinically indicated. The Social Affect (SA) score (10 items; range 0–20), the Restricted and Repetitive Behavior (RRB) score (4 items; range 0–8), the overall score (sum of the SA and RRB scores) and the Calibrated Severity Score (CSS; Gotham et al., 2009) (range 1–10) were used to describe the sample, with higher scores indicating more severe ASD symptoms. All ADOS-2 were administered by research assistants or a senior clinical psychologist trained to research reliability level. ADOS-2 were videotaped and the recordings were used for this study (see below).

#### Autism Diagnostic Interview-Revised Interview

The Autism Diagnostic Interview-Revised interview (ADI-R; Rutter et al., 2003) is a standardized semi-structured interview used in the diagnosis of ASD. It is designed for use with a parent or caregiver who is familiar with the developmental history and current behavior of individuals older than 2 years. The diagnostic algorithms rely on scores derived for 3 major developmental domains (language and communication, reciprocal social interaction, and restricted, repetitive, and stereotyped behaviors and interests) and for a fourth criterion establishing evidence of first developmental abnormalities before age 3. Only caregivers of the ASD group were interviewed with the ADI-R. Interviews were administered by trained interviewers. Data were reviewed by the diagnostic team and integrated in the best estimate clinical procedures used to confirm diagnoses.

#### Social Responsiveness Scale

The SRS was designed to measure autistic symptomatology and traits, and the severity of the associated social impairment (Constantino, 2012). It is applicable to 4-to 18-year-old and can be completed in about 15–20 min by a parent or any other informant knowledgeable about the child’s behavior across contexts and over time. The SRS comprises 65 items each scored on a Likert scale ranging from 1 (not true) to 4 (almost always true), with 17 items being reverse-scored. Using data from a general population non-clinical sample, t-scores derived from the U.S. population can be employed for individual testing and clinical interpretation. Total t-scores were used to describe the sample.

#### Intellectual Level

Intellectual level of participants was estimated with a short form of the Wechsler Intelligence Scale for Children -4th Edition (Wechsler, 2003). Three subtests were administered: Information Block Design and Vocabulary allowing a full-scale IQ to be estimated from the sum of scaled scores of the three subtests according to formula set out by Sattler and Dumont (2004).

#### Language Profile

Language skills and linguistic pragmatic abilities were assessed using the parent- completed Children Communication Checklist second edition (CCC-2; Bishop, 2013). CCC-2 is a widely used 70-items standardized checklist of pragmatic and social communication behaviors applicable to children aged 4–17 years. Caregivers are asked to make a frequency judgment about how often behaviors occur on 4-point scale (0, less than once a week; 1, at least once a week; 2, once or twice a day; 3, several times a day). CCC-2 is divided in 10 subscales (7 items each, including 5 weaknesses and 2 strengths items) measuring: (A) speech, (B) syntax, (C) semantics, (D) coherence, (E) inappropriate initiation, (F) stereotyped language, (G) the use of context, (H) non-verbal communication, (I) social relationships and (J) interests. Each subscale raw score is converted to age-standardized scores (mean = 10; SD = 3). A General Communication Composite (GCC) is derived by summing scores from scales A to H (mean = 100; SD = 15). The Social Interaction Difference Index (SIDI) score is calculated as the difference between the sum of the four pragmatics (E through H) and the 4 structural (A through D) language subscores, with more negative values indicative of autism.

#### Data: Speech Samples

In this study, we used ADOS-2 recordings from 4 tasks that are conversational in nature: “emotions conversation” (EC), “social difficulties and annoyance conversation” (SDAC), “friends and marriage conversation” (FMC), and “loneliness conversation” (LC). These four tasks occur in the second half of the ADOS-2; they do not require objects, books or images, and are purely conversational. The focus is on the understanding by the participant of the nature of emotions and social relationships. The examiner uses some preset interview questions that are open-ended and designed to facilitate the flow of conversation. Follow-up probes are discretionarily used by the examiner to maintain that flow.

### Data Analysis

#### Harmonic Model

Voiced speech is a quasi-periodic signal with slowly time-varying amplitudes and harmonically related frequencies. Thus, a HM is a suitable choice to characterize voiced speech (Stylianou, 2001). This model decomposes the voiced speech into a periodic component and a non-periodic component related to noise. The periodic component is modeled by a weighted combination of sines and cosines terms (harmonic terms), with frequencies that are multiples of the fundamental frequency (i.e., pitch). Analyzing the noise-free periodic component of speech increases the chance of more accurate pitch estimates for monophonic sounds. This model is tailored to capture the rich harmonic nature of voiced segments in speech and has applications in speech synthesis, voice conversion, speech enhancement, and speech coding.

#### Model Expression

Adopting notations from Stylianou (2001), we express the model as follow. Let *y* = [y(t_1_), y(t_2_),…, y(t*_*N*_*)]*T* denote the N speech samples in a voiced frame, measured at times t_1_, t_2_,…, t*_*N*_.* The samples can be represented with a HM with an additive noise, *n* = [n(t_1_), n(t_2_),…, n(t*_*N*_*)]*T*, modeled by a Gaussian distribution [N (*μ*, *σ_*n*_*^2^)] as follows:

(1) $$s\,\left(t\right)=a_{0}+\sum_{h=1}^{H}\cos\left(2\,\pi \,{\text{f}}_{0}\,h\,t\right)+b_{h}\,\sin\left(2\,\pi \,f_{0}\,h\,t\right)$$

(2) y⁢(t)=s⁢(t)+n⁢(t)

where *H* denotes the number of harmonics and 2*πf*_0_ stands for the fundamental angular frequency. The harmonic signal can be factorized into harmonic components that include coefficients of sinusoidal functions, α_*h*_, β_*h*_, the angular frequency, 2*πf*_0_, and the model order, *H*. Assuming the noise component is Gaussian, unknown parameters of the model ([*a*_0_, α_*h*_, β_*h*_, *f*_0_, σ_*n*_^2^, *H*]) can be estimated using a maximum likelihood (ML) estimation method (Tabrikian et al., 2004). However, the straightforward application of this model leads, too, to “octave errors” (Asgari and Shafran, 2013). In our prior work, we mitigated these errors by modifying the HM using a local smoothing function while estimating the pitch candidates (Asgari and Shafran, 2013) and reformulating the parameter estimation using a maximum *a posteriori* probability (MAP) framework to prevent overfitting due to the model complexity (Asgari and Shafran, 2018). Our experimental results showed a significant improvement in accuracy of pitch detection against three widely used PDAs (de Cheveigné and Kawahara, 2002; Sun, 2002; Kawahara et al., 2008), especially in adverse noisy conditions (Asgari and Shafran, 2013).

#### Speech Processing

To automatically extract acoustic/prosodic measures of speech samples, we first found and grouped those speech segments on each conversational activity that belong to a given participant. This created an audio profile of four conversational activities, each consisting of participant’s speech segments. The length of speech segments varied across participants depending upon the content and the length of the conversation. Next, we represented voice characteristics of each participant with a global feature vector of acoustic and prosodic measures using speech processing algorithms. Our speech analysis framework comprises a cascade of three *short-term*, *segment-level*, and *subject-level* analyses of the speech signal as described in the next section.

#### Short-Term Speech Analysis

According to the articulatory model of speech production (Deng, 1999), speech signal is inherently a non-stationary process and its characteristics vary over time; therefore, it cannot be analyzed by common digital signal processing (DSP) methods such as Fast Fourier transform (FFT) algorithm (Proakis and Manolakis, 1988). However, as vocal folds slowly move relative to the frequency of voice signal, the voice properties can be assumed stationary in short period of times (e.g., 10 ms) commonly known as a short-term *frame*. Assuming the short-term stability of voice over 10 ms long frames, we first sliced every segment of speech into 25 ms long overlapping frames at a rate of 100 frames per second. Next, we removed silence parts from the speech segment using an energy-based silence detection algorithm. Then, using the HM of speech, we detected voiced and unvoiced speech frames and subsequently extracted the four quantities from voiced frames related to harmonic content: pitch, jitter, shimmer, and harmonic-to-noise ratio (HNR). Pitch-related statistics convey considerable information about the emotional state of speakers (Busso et al., 2009). We used the HM of speech for detecting pitch candidates as described in our prior work (Asgari and Shafran, 2013). Note that pitch variations are inherently limited by the motion of the articulators in the mouth during speech production; hence, they cannot vary arbitrarily between adjacent frames. We enforced a smoothness constraint on successive frames of a speech segment using a first order Markov model and detected the pitch contour over the segment using a Viterbi algorithm (Asgari and Shafran, 2013). The refined version of HM, known as TV-HM, allows the amplitude of the harmonics to vary smoothly over the duration of the frame and thus it is able to follow perturbations associated with shimmer and jitter. We used TV-HM to quantify cycle-to-cycle frequency (jitter) and amplitude (shimmer) variations in pitch frequency. Finally, we augmented our acoustic/prosodic measures with HNR derived from the HM. We refer the reader to our prior work (Asgari and Shafran, 2013) for more computational detail on extracting these measures. Those measures derived from HM and TV-HM were combined with the following standard speech measures computed across both voiced and unvoiced frames: (1) loudness that measures the amount of speaking volume (energy) based on the square root of average of squared value of the signal’s amplitude; (2) cepstral coefficients that are widely used to characterize the dynamics of speech articulation. Shape of the spectral envelop is extracted from cepstral coefficients. Thirteen cepstral coefficients of each frame were augmented with their first- and second-order time derivatives; and, (3) spectral entropy that produce useful proxy for cues related to voicing quality. Spectral entropy can be used to characterize “speechiness” of the signal and has been widely employed to discriminate speech from noise. Therefore, we computed the entropy of the log power spectrum for each frame, where the log domain was chosen to mirror perception.

#### Segmental Speech Measures

The short-term measures computed at the frame-level were summarized into a feature vector of fixed dimension for each speech segment sliced from a conversation. Features extracted from voiced regions tend to differ in nature compared to those from unvoiced regions. To preserve these differences, we separately summarized each frame-level measure across all frames from the voiced and unvoiced segments in terms of standard distribution statistics such as mean, median, variance, minimum and maximum. The resulting segment-level voice quality and prosody feature vector was later augmented by duration-related statistics. Duration and frequency characteristics of speech provide useful cues about speaking rate and fluency (Healey and Adams, 1981; Andrews et al., 1982) and were computed based on the number and duration of voiced and unvoiced segments.

#### Subject-Level Speech Measures

The frequency of speech segments collected over the course of four ADOS-2 conversational activities, was different across participants. For the purpose of training machine learning models, we needed to summarize segment-level feature vectors into a global feature vector of a fixed dimension across all participants. Using the same statistical function used for summarizing frame-level measures, we summarized segment-level feature vectors of each conversational activity into an activity-level feature vector. Finally, we augmented four activity-level feature vectors and constructed a global subject-level feature vector representing the voice characteristics of a participant.

### Statistical Analysis

The utility of extracted acoustic/prosodic measures was evaluated using machine learning algorithms in detection of ASD from TD group. From an open-source toolkit, Scikit-learn (Pedregosa et al., 2011), we adopted a support vector machine (SVM) model for our classification task. All experimental results, presented in the next sections, were based on the linear SVM as it outperformed the non-linear SVM. We also used a L1-norm regularization term that is well-known in applications requiring sparse solutions, assigning zero values to useless regression coefficients (Tibshirani, 1996). Additionally, we repeated the experiment using a “Chance” classifier which randomly assigned participants into ASD and TD classes. Prior to training a SVM model, we scaled the range of computed features into a constrained range using Scikit-learn’s *RobustScaler*. This step was necessary in our computational framework as we noticed that the range of derived features greatly differed from each other.

#### Evaluation Metrics

To evaluate the performance of the proposed classifier, we computed the sensitivity and specificity of detecting ASD using the receiver operating characteristic (ROC) approach. Area under the curve (AUC) of ROC were compared across SVM models. We also calculated the %95 confidence intervals of the area under the curve of receiver operating characteristics (AUC-ROC) for a meaningful comparison between classifiers. To validate results and establish their independence from our specific data sets, and also to reduce the overfitting problem, we used five-fold cross-validation (CV) techniques (Kohavi, 1995) in which the training, development, and test sets are rotated over the entire data set. With this approach, the optimal parameters of SVM models were only learned from the training examples (four out of five sets), totally blinded from the test examples, and the fifth one only used for reporting the performance estimates.

#### Imbalanced Data

A common issue often encountered in a binary classification task raising a concern on the validity of evaluation results is disproportionate distribution of training examples amongst classes. We tackled this potential issue through an iterative process using an under-sampling technique. At each iteration, we first randomly drew 28 samples from the majority class (ASD) to match the sample size with the TD class. Next, we evaluated the five-fold CV on the matched samples and accumulated averaged scores across test folds. This iteration repeated until the overall performance converged to a steady state. Our experiments showed that randomness effects were reduced by repeating the process until average scores across all iterations had converged after about 100 times.

### Ethical Approval

This study was reviewed and approved by the Institutional Review Board of OHSU.

## Results

Sample characteristics are summarized in Table 1. There was no statistically significant difference for age, race, and ethnicity between the groups. As predicted, all autism measures (ADOS-2, SRS) differed significantly between groups. Participants with ASD had significantly lower language and IQ scores than controls although the mean IQ for the ASD group was close to the population mean. Voice-related abnormalities of ASD can be associated with different dimensions of speech. To understand the contribution of the different measures, we broadly categorized them into two groups: prosodic and articulation. The group of features included pitch, jitter, shimmer, HNR, and loudness, and the articulation group was derived from spectral entropy and cepstral coefficients. For this purpose, speech prosodic measures were extracted from all ADOS-2 activities. We then independently trained three SVM classifiers using subject-level feature vectors constructed from these two groups of measures (prosody, articulation, and both). Table 2 reports the performance of SVM models measured in terms of averaged Sensitivity, Specificity, and AUC-ROC over 100 repetitions of CV. Note that speech measures in these experiments were extracted from all ADOS-2 activities. The results indicate that prosodic features more accurately distinguished ASD subjects from those with TD in comparison to features of articulation due to better specificity of prosody over articulation. Combining both measures also improved overall accuracy through an additional gain of specificity whereas sensitivity remained constant, similar to that of prosody or articulation alone. Due to the specificity of articulation measures, we only used prosodic measures for the rest of our experiments.

**TABLE 1:** Sample characteristics.

	**ASD (*N* = 90)**	**TD (*N* = 28)**	***P*-value**
Male sex, *N*(%)	75 (80.6)	12 (40.0)	<0.001
Age in years, *X*(SD)	10.84 (2.20)	10.96 (1.54)	0.807
range	7.8, 15.3	7.0, 15.0	
Hispanic, *N*(%)	13 (14.4)	5 (18)	0.768
Race white, *N*(%)	80 (86.0)	24 (85.7)	1.000
**Clinical profiles**			
WISC-IV full scale IQ	99.0 (20.00)	113.4 (12.3)	0.005
ADOS-2 total SA score	9.40 (3.57)	1.04 (1.86)	<0.001
ADOS-2 total RRB score	3.59 (1.56)	0.52 (0.71)	<0.001
ADOS-2 total score	12.99 (3.50)	1.56 (2.29)	<0.001
ADOS-2 CSS score	7.55 (1.44)	1.22 (1.04)	<0.001
SRS total t-score	77.35 (10.93)	43.96 (4.14)	<0.001
**Language scores**			
CCC2 GCC	45.49 (15.28)	91.89 (8.48)	<0.001
CCC2 SIDI	−7.51 (9.13)	3.07 (5.14)	<0.001

*ADOS-2, Autism Diagnosis Observational Schedule; SA, Social Affect; RRB, Restricted Repetitive Behavior; CSS, Calibration Severity Score; SRS, Social Responsiveness Scale; CCC2, Children’s Communication Checklist, 2nd; GCC, General Communication Composite of the CCC2; SIDI, Social Interaction Difference Index of the CC.*

**TABLE 2:** Diagnostic classification derived from speech prosodic or articulation features, and their combination.

**Speech Measures**	**ROC AUC**	**Sensitivity**	**Specificity**	**Accuracy**
Prosodic	82.23% (81.11%,83.35%)	69.67%	76.83%	73.30%
Articulation	67.98% (66.62%,69.35%)	62.63%	62.53%	62.58%
Prosodic + Articulation	78.52% (77.30%,79.73%)	69.43%	70.74%	70.02%
Chance	49.90% (48.17%,51.63%)	50.13%	50.87%	49.63%

*ROC, receiver operating characteristics; AUC: area under the curve.*

### Effectiveness of ADOS-2 Activities

In our initial experiment, we concatenated subject-level feature vectors extracted from all ADOS-2 conversational activities for learning SVM models. The four types of ADOS-2 conversational activities evoke different emotional states and may translate into varied speech/voice outputs. To examine the influence of conversational content, we extracted our prosodic measures separately from each activity and using those, we trained four classification models. The results are reported in Table 3 for the SVM model with the linear kernel and L1-norm regularization term. Comparing the AUC-ROC of SVM classifiers, it is observed that prosodic measures extracted from “FMC” more strongly differentiated ASD from TD participants in comparison to other ADOS-2 activities. FMC achieved overall better accuracy due to superior specificity. When examined together, the combination of prosodic measures across two tasks (FMC and EC) achieved the best overall accuracy with comparable and satisfactory levels of both sensitivity and specificity. However, when compared to FMC alone, overall accuracy was similar; FMC alone would provide an adequate sampling context and could be selected in circumstances where reducing the false positive rate is required.

**TABLE 3:** Prosodic measures performance in predicting diagnosis, with four ADOS-2 tasks and their combination.

**ADOS-2 activity**	**ROC AUC**	**Sensitivity**	**Specificity**	**Accuracy**
FMC	83.04% (82.00%,84.09%)	70.02%	79.17%	74.52%
EC	81.63% (80.51%,82.76%)	70.82%	75.28%	73.05%
SDAC	81.21% (80.06%,82.37%)	70.39%	72.50%	71.35%
LC	78.15% (76.97%,79.34%)	64.97%	74.90%	69.82%
FMC + EC	82.69% (81.63%,83.76%)	70.20%	77.35%	73.82%
FMC + EC + LC	81.92% (80.77%,83.07%)	70.52%	76.85%	73.64%
FMC + EC + LC + SDAC	81.65% (80.52%,82.77%)	70.23%	76.41%	73.42%

*ADOS-2, Autism Diagnosis Observational Schedule; ROC, receiver operating characteristics; AUC, area under the curve; FMC, friends and marriage conversation; EC, emotions conversation; SDAC, social difficulties and annoyance conversation; LC, loneliness conversation.*

*Correlations between subject-level prosodic features (pitch and loudness) to clinical ratings of language profile and autism severity.*

As the samples in above classification tasks were different in terms of subjects’ IQ level (see Table 1), we repeated these analyses on a sub-sample of 28 subjects with ASD closely matched for IQ with 28 TD controls. In these 28 matched pairs (mean IQ = 113.4; *p*-value = 0.98), a similar pattern of results was obtained for FMC with respect to both overall accuracy (77.6%) and discriminant ability (ROC AUC = 88.27%).

### Most Informative Prosodic Measures

Not all the extracted measures are expected to be useful, and in fact many are likely to be noisy. From the Scikit-learn toolkit (Pedregosa et al., 2011), we chose a feature selection technique known as recursive feature elimination with cross-validation (RFECV), which ranks the importance of features based on a given scoring function and returns a subset of optimal features. Applying the RFECV method, we evaluated several models trained on a subset of prosodic features. Experimental results revealed that the combination of pitch and loudness measures constructed the optimal subset of prosodic measures in this ASD vs. TD classification task. To further investigate the relationship between these two measures (pitch and loudness) and clinical ratings of the subjects, we computed partial Spearman correlation coefficients controlling for WISC IQ scores (Table 4). Both pitch and loudness had a consistent significant association with autism severity when assessed by independent informants (professional for the ADOS-2 and parent for SRS). The prosodic measures showed a moderate correlation with CCC2 language scores although loudness was unrelated to the SIDI score (*r* = −0.170, *p* = 0.13) that measures pragmatic problems specifically.

**TABLE 4:** Correlations^*a*^ between subject-level prosodic features (pitch and loudness) to clinical ratings of language profile and autism severity.

**Prosodic Measures**	**CCC2 – GCC**	**CCC2- SIDI score**	**ADOS-2 CSS**	**SRS-T-score**
Pitch	**0.274**	**0.295**	**−0.312**	*−0.264*
Loudness	**−0.386**	−0.170	**0.324**	**0.323**

*^*a*^Non-parametric partial Spearman coefficients (adjusted on WISC IQ scores); italics indicate 0.05 < *p* < 0.10; bold indicate 0.01 < *p* < 0.05; bold and underlined indicate *p* < 0.01.*

*CCC2, Children’s Communication Checklist, 2nd; GCC, General Communication Composite of the CCC2; SIDI, Social Interaction Difference Index of the CC; ADOS-2, Autism Diagnosis Observational Schedule; CSS, Calibration Severity Score; SRS, Social Responsiveness Scale.*

**FIGURE 1:**
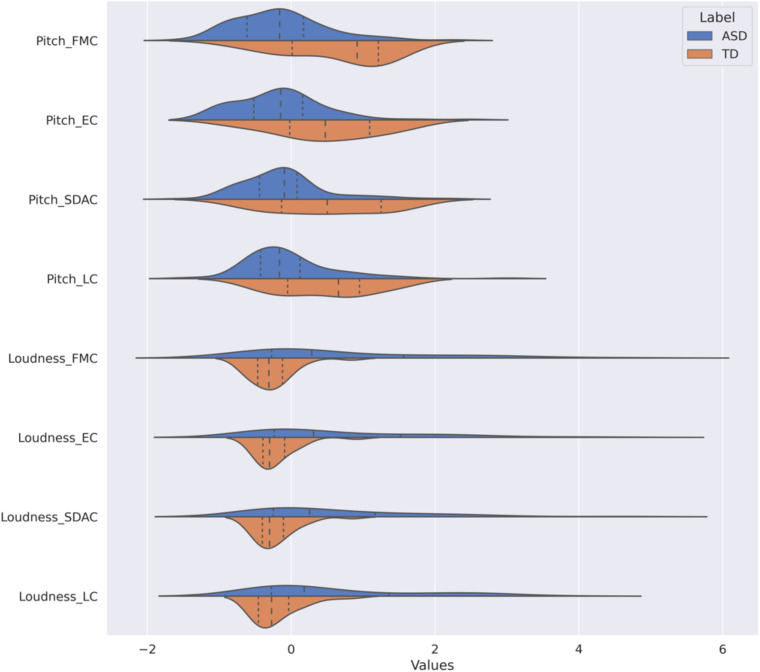
Distribution of pitch and loudness measures across four Autism Diagnosis Observational Schedule (ADOS-2) activities, by clinical group. The name in *y*-axis combines both feature name and the name of ADOS-2 activity in the format “{feature name}_{ADOS-2 activity name}.” We have two features: pitch and loudness. The ADOS-2 activities are friends and marriage conversation (FMC), emotions conversation (EM), social difficulties and annoyance conversation (SDAC), and loneliness conversation (LC). Two diagnosis labels are included: typically developing (TD) and autism spectrum disorder (ASD). The dynamic range of features have been normalized according to the *RobustScaler* approach. The dotted lines from left to right indicate 25, 50, and 75% quantiles. The dynamic range of features have been normalized according to the *RobustScaler* approach. The dotted lines from left to right indicate 25, 50, and 75% quantiles. ADOS-2, Autism Diagnosis Observational Schedule; ASD, autism spectrum disorder; TD, typically developing; F0, pitch; RMS, loudness; FMC, friends and marriage conversation; EC, emotions conversation; SDAC, social difficulties and annoyance conversation; LC, loneliness conversation.

**FIGURE 2:**
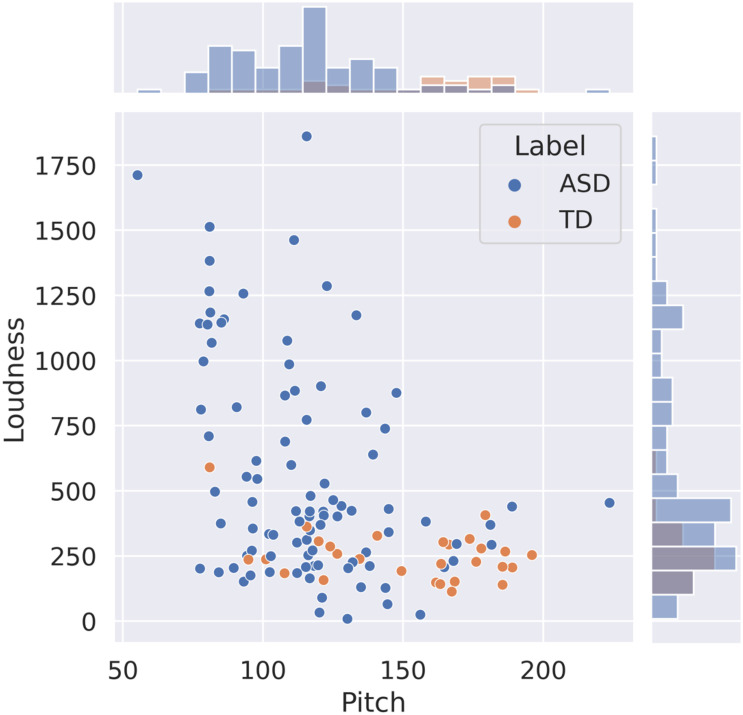
Visualization of two-dimensional feature space (pitch and loudness) for “friends and marriage conversation” activity. The histogram on *x*-axis shows the value distribution of pitch and the one on *y*-axis is the distribution of loudness. Two diagnosis labels are included: TD and ASD. ASD, autism spectrum disorder; TD, typically developing.

Probability distributions of the pitch and loudness features, depicted in Figure 1, show that the pitch values are lower in ASD participants than in controls but that the dispersion is comparable across the 2 groups. In contrast, the loudness means in ASD subjects exceeded those in TD subjects across all ADOS-2 tasks with much larger variability. We further looked at combination pitch and loudness features across both ASD and TD classes. The resulting 2-dimensional visualization extracted from the optimal sampling context, i.e., FMC, is shown in Figure 2. As it is observed in this plot, two separate clusters of ASD and TD subjects can be noticed and participants with ASD are associated with lower pitch value with wider loudness range compared to those with TD. The plot also indicated the discriminatory power of combined pitch and loudness measures in discriminating ASD from TD subjects.

## Discussion

Acoustic and prosodic aspects of speech have been shown to be biomarkers of ASD (Fusaroli et al., 2017). However, most speech processing algorithms are not suitable for characterizing these biomarkers as they do not have the necessary time-frequency resolution to capture the fine fluctuations observed in impaired speech. In this proof of concept study, we proposed automated methods for characterizing the abnormal prosodic pattern of autism that succeeded in distinguishing subjects with ASD from TD controls. Our proposed method is novel and utilizes pitch as a key feature in describing atypical prosodic and vocal features. Pitch-related features play an important role in characterizing pathologies such as Parkinson’s disease, clinical depression and autism spectral disorder (ASD). Of the available pitch estimators, only the HM exploits the inherent harmonic structure of the voiced speech and has model parameters which can be estimated in an unsupervised manner without the need to learn a model on training data. We recently developed a TV-HM of speech (Asgari and Shafran, 2018) to address a few drawbacks of the HM and demonstrated improvements in accuracy and robustness of pitch estimation over several pitch trackers, including those employed in similar studies for characterizing abnormal prosody of autistic children (Bonneh et al., 2011; Santos et al., 2013). Using TV-HM of speech, we extracted a set of voice measures of natural speech samples (NSSs) to capture atypical patterns of autistic speech and voice that can discriminate children with ASD from those with TD. Dividing our proposed speech measures into two sets of prosodic and articulation measures, we examined the efficacy of each set in distinguishing ASD from TD controls. The experimental results showed the superiority of prosodic measures in comparison to those that characterize the articulation features. In keeping with inconsistent results from previous studies (e.g., Shriberg et al., 2001), articulation measures alone only achieved a modest AUC-ROC curve (≈68%). By contrast, prosodic measures achieved a higher level of AUC-ROC (≈82%) that rose to 83% when the voice sampling context was optimized. This confirms results from several prior studies of prosodic aspects in ASD where overall accuracy levels in multivariate models were comparable (Bonneh et al., 2011; Fusaroli et al., 2017).

Additionally, we separately extracted our voice-based measures from each ADOS-2 activity in order to examine their influence in the classification tasks. Results showed that the ADOS-2 “FMC” task is better than three other ADOS-2 tasks in distinguishing subjects with ASD. This pattern of differential performance according to voice sampling context is important to appreciate. Prior studies of pitch have shown some inconsistencies due to the variability of methods used across investigations. One source of heterogeneity was the voice sampling conditions that varied from natural recordings at home (Oller et al., 2010; Gong et al., 2018), with or without structured social interactions with caregivers or other partners, or recorded professional evaluations employing tasks with varying social and cognitive demands (Bonneh et al., 2011; Santos et al., 2013) that in turn affected the pitch discriminant ability. Finally, we employed a feature selection method in order to pick out the most informative prosodic measures. Our results showed that the combination of pitch and loudness measures resulted in the best performance. Further analysis showed associations between pitch and loudness with language scores and autism severity, a noticeable result since it was established with separate informants. Despite the small sample size available for this pilot analysis, we found highly significant differences indicating that the magnitude of differences between groups was very large which is very encouraging for future research.

These results were not adjusted for multiple comparisons and should be viewed as preliminary. They require replication in larger samples, more balanced with respect to IQ and gender composition, that we are planning to perform in the near future. Nevertheless, several research implications of our findings and future directions are worth noting. First, using natural language processing approaches, our group has generated several automatic discourse measures that, when applied to transcripts of ADOS-2 in the same sample have shown an ability to differentiate youth with ASD from controls, both in isolation and when taken in combination (Salem et al., 2021). A logical next step will be for us to compare the levels of accuracy that can be achieved, on the same subjects, by voice analysis or language analysis only, and to evaluate if combining voice and language analysis would result in gains of accuracy of predicting diagnostic status. Preliminary evidence from a prior study (Gong et al., 2018) that analyzed acoustic and linguistic features simultaneously suggested that acoustic measures were more powerful than linguistic ones to discriminate between ASD and controls. Second, there is a recognized dearth of outcome measures available for treatment research in autism (Anagnostou et al., 2015). The methodologies that we are using to detect atypical voice and language patterns are easy to deploy, user-friendly, objective and cost-effective, and they can be implemented in natural settings and repeated often without risking jeopardizing their validity. Moreover, new tools have recently been made available through smart phone and other technologies (Xu et al., 2014; Yatawatte et al., 2016) that provide inexpensive and reliable ways to collect large amounts of data in naturalistic settings. To qualify for measures of outcome in treatment studies, it is necessary to demonstrate their ability to reliably capture change which could be demonstrated in longitudinal studies (Schoen et al., 2011) or ongoing randomized clinical trials. Third, as was already explored previously (Paul et al., 2011; Schoen et al., 2011; Santos et al., 2013) use of voice analysis in preverbal children at risk of developing autism could be beneficial to early detection in preverbal toddlers. Likewise, identification of atypical voice patterns might enhance diagnostic procedures in young children with minimal or no language.

## Data Availability Statement

The raw data supporting the conclusions of this article will be made available by the authors, upon request to the authors.

## Ethics Statement

The studies involving human participants were reviewed and approved by OHSU IRB. Written informed consent to participate in this study was provided by the participants’ legal guardian/next of kin.

## Author Contributions

All authors equally contributed in design, execution, and evaluation of experiments in addition to drafting the manuscript. All authors revised the manuscript and approved the final version prior to submission.

## Conflict of Interest

The authors declare that the research was conducted in the absence of any commercial or financial relationships that could be construed as a potential conflict of interest.

## Publisher’s Note

All claims expressed in this article are solely those of the authors and do not necessarily represent those of their affiliated organizations, or those of the publisher, the editors and the reviewers. Any product that may be evaluated in this article, or claim that may be made by its manufacturer, is not guaranteed or endorsed by the publisher.

## References

[B1] American Psychiatric Association (2013). *Diagnostic and Statistical Manual of Mental Disorders*, 5th Edn. Washington, DC: American Psychiatric Association.

[B2] Anagnostou E. Jones N. Huerta M. Halladay A. K. Wang P. Scahill L. (2015). Measuring social communication behaviors as a treatment endpoint in individuals with autism spectrum disorder. *Autism* 19, 622–636. 10.1177/1362361314542955 25096930

[B3] Andrews G. Howie P. M. Dozsa M. Guitar B. E. (1982). Stuttering: speech pattern characteristics under fluency-inducing conditions. *J. Speech Lang. Hear. Res.* 25 208–216. 10.1044/jshr.2502.208 7120960

[B4] Asgari M. Shafran I. (2010a). “Extracting cues from speech for predicting severity of parkinson’s disease,” in *Proceedings of the 2010 IEEE International Workshop on Machine Learning for Signal Processing, Kittila*, (Piscataway, NJ: IEEE), 462–467. 10.1109/MLSP.2010.5589118 PMC7924985 33659095

[B5] Asgari M. Shafran I. (2010b). “Predicting severity of Parkinson’s disease from speech,” in *Proceedings of the 2010 Annual International Conference of the IEEE Engineering in Medicine and Biology, Buenos Aires*, (Piscataway, NJ: IEEE), 5201–5204. 10.1109/IEMBS.2010.5626104 PMC7889280 21095825

[B6] Asgari M. Shafran I. (2013). “Improving the accuracy and the robustness of harmonic model for pitch estimation,” in *Proceedings of the 14th Annual Conference of the International Speech Communication Association*, Lyon, 1936–1940.

[B7] Asgari M. Shafran I. (2018). Improvements to harmonic model for extracting better speech features in clinical applications. *Comput. Speech Lang.* 47 298–313. 10.1016/j.csl.2017.08.005

[B8] Asgari M. Shafran I. Sheeber L. B. (2014). “Inferring clinical depression from speech and spoken utterances,” in *Proceedings of the 2014 IEEE International Workshop on Machine Learning for Signal Processing (MLSP), Reims*, (Piscataway, NJ: IEEE), 1–5. 10.1109/mlsp.2014.6958856 PMC7719299 33288990

[B9] Bishop D. (2013). *Children’s Communication Checklist (CCC-2).* Berlin: Springer.

[B10] Boersma P. Weenink D. (2001). *Praat Speech Processing Software.* Amsterdam: Institute of Phonetics Sciences of the University of Amsterdam.

[B11] Bonneh Y. S. Levanon Y. Dean-Pardo O. Lossos L. Adini Y. (2011). Abnormal speech spectrum and increased pitch variability in young autistic children. *Front. Hum. Neurosci.* 4:237. 10.3389/fnhum.2010.00237 21267429 PMC3024839

[B12] Busso C. Lee S. Narayanan S. (2009). Analysis of emotionally salient aspects of fundamental frequency for emotion detection. *IEEE Trans. Audio Speech Lang. Process.* 17 582–596. 10.1109/tasl.2008.2009578

[B13] Constantino J. Gruber C. (2005). *Social Responsive scale (SRS) Manual.* Los Angeles, CA: Western Psychological Services.

[B14] Constantino J. N. (2012). *Social Responsiveness Scale: SRS-2.* Torrance, CA: Western Psychological Services.

[B15] Cooper K. L. Hanstock T. L. (2009). Confusion between depression and autism in a high functioning child. *Clin. Case Stud.* 8 59–71. 10.1177/1534650108327012

[B16] de Cheveigné A. Kawahara H. (2002). YIN, a fundamental frequency estimator for speech and music. *J. Acoustical Soc. Am.* 111 1917–1930. 10.1121/1.1458024 12002874

[B17] Deng L. (1999). *Computational Models for Speech Production. Computational Models of Speech Pattern Processing.* Berlin: Springer, 199–213.

[B18] Drugman T. Alwan A. (2011). “Joint robust voicing detection and pitch estimation based on residual harmonics,” in *Proceedings of the 12th Annual Conference of the International Speech Communication Association*, Florence.

[B19] Fusaroli R. Lambrechts A. Bang D. Bowler D. M. Gaigg S. B. (2017). Is voice a marker for autism spectrum disorder? A systematic review and meta-analysis. *Autism Res.* 10 384–407. 27501063 10.1002/aur.1678

[B20] Gong Y. Yatawatte H. Poellabauer C. Schneider S. Latham S. (2018). “Automatic autism spectrum disorder detection using everyday vocalizations captured by smart devices,” in *Proceedings of the 2018 ACM International Conference on Bioinformatics, Computational Biology, and Health Informatics*, 465–473.

[B21] Gotham K. Pickles A. Lord C. (2009). Standardizing ados scores for a measure of severity in autism spectrum disorders. *J. Autism Dev. Disord.* 39 693–705. 10.1007/s10803-008-0674-3 19082876 PMC2922918

[B22] Healey E. C. Adams M. R. (1981). Speech timing skills of normally fluent and stuttering children and adults. *J. Fluency Disord.* 6 233–246. 10.1016/0094-730x(81)90004-8

[B23] Hönig F. Batliner A. Weilhammer K. Nöth E. (2010). “Automatic assessment of non-native prosody for english as L2,” in *Proceedings of the Speech Prosody 2010-5th International Conference.*

[B24] Kawahara H. Morise M. Takahashi T. Nisimura R. Irino T. Banno H. (2008). “Tandem-straight: a temporally stable power spectral representation for periodic signals and applications to interference-free spectrum, F0, and aperiodicity estimation,” in *Proceedings of the 2008 IEEE International Conference on Acoustics, Speech and Signal Processing, Las Vegas, NV*, (Piscataway, NJ: IEEE), 3933–3936.

[B25] Kohavi R. (1995). “A study of cross-validation and bootstrap for accuracy estimation and model selection,” in *Proceedings of the 14th international joint conference on Artificial intelligence – August 1995*, Vol. 14 Montreal, QC, 1137–1145.

[B26] Kumaraswamy B. Poonacha P. (2019). Octave error reduction in pitch detection algorithms using fourier series approximation method. *IETE Tech. Rev.* 36 293–302. 10.1080/02564602.2018.1465859

[B27] Lord C. Rutter M. DiLavore P. C. Risi S. (2003). *Autism Diagnostic Observation Schedule: ADOS.* Los Angeles, CA: Western Psychological Services.

[B28] Loveall S. J. Hawthorne K. Gaines M. (2021). A meta-analysis of prosody in autism, williams syndrome, and down syndrome. *J. Commun. Disord.* 89:106055. 10.1016/j.jcomdis.2020.106055 33285421

[B29] Mubashir S. Farrugia M. Coretti L. Pessia M. D’Adamo M. C. (2020). *Autism Spectrum Disorder.*

[B30] Oller D. K. Niyogi P. Gray S. Richards J. A. Gilkerson J. Xu D. (2010). Automated vocal analysis of naturalistic recordings from children with autism, language delay, and typical development. *Proc. Natl. Acad. Sci. U.S.A.* 107, 13354–13359. 10.1073/pnas.1003882107 20643944 PMC2922144

[B31] Patel S. P. Nayar K. Martin G. E. Franich K. Crawford S. Diehl J. J. (2020). An acoustic characterization of prosodic differences in autism spectrum disorder and first-degree relatives. *J. Aut. Dev. Disord.* 50, 3032–3045. 10.1007/s10803-020-04392-9 32056118 PMC7374471

[B32] Paul R. Fuerst Y. Ramsay G. Chawarska K. Klin A. (2011). Out of the mouths of babes: vocal production in infant siblings of children with ASD. *J. Child Psychol. Psychiatry* 52, 588–598. 21039489 10.1111/j.1469-7610.2010.02332.x PMC3078949

[B33] Paul R. Shriberg L. D. McSweeny J. Cicchetti D. Klin A. Volkmar F. (2005). Brief report: relations between prosodic performance and communication and socialization ratings in high functioning speakers with autism spectrum disorders. *J. Autism Dev. Disord.* 35 861. 10.1007/s10803-005-0031-8 16283080

[B34] Pedregosa F. Varoquaux G. Gramfort A. Michel V. Thirion B. Grisel O. (2011). Scikit-learn: machine learning in python. *J. Mach. Learn. Res.* 12 2825–2830.

[B35] Proakis J. G. Manolakis D. G. (1988). *Introductionto Digital Signal Processing (Prentice Hall Professional Technical Reference).*

[B36] Rutter M. Le Couteur A. Lord C. (2003). *Autism Diagnostic Interview-Revised*, Vol. 29. Los Angeles, CA: Western Psychological Services, 30.

[B37] Salem A. C. MacFarlane H. Adams J. R. Lawley G. O. Dolata J. K. Bedrick S. (2021). Evaluating atypical language in autism using automated language measures. *Sci Rep.* 11:10968. 10.1038/s41598-021-90304-5 34040042 PMC8155086

[B38] Santos J. F. Brosh N. Falk T. H. Zwaigenbaum L. Bryson S. E. Roberts W. (2013). “Very early detection of autism spectrum disorders based on acoustic analysis of pre-verbal vocalizations of 18-month old toddlers,” in *Proceedings of the 2013 IEEE International Conference on Acoustics, Speech and Signal Processing* (IEEE), 7567–7571.

[B39] Sattler J. Dumont R. (2004). *Assessment of Children: WISC-IV and WPPSI-III Supplement.* San Diego, CA: Jerome M. Sattler Publisher Inc.

[B40] Schoen E. Paul R. Chawarska K. (2011). Phonology and vocal behavior in toddlers with autism spectrum disorders. *Aut. Res.* 4, 177–188. 10.1002/aur.183 21308998 PMC3110574

[B41] Sharda M. Subhadra T. P. Sahay S. Nagaraja C. Singh L. Mishra R. (2010). Sounds of melody—pitch patterns of speech in autism. *Neurosci. Lett.* 478 42–45. 10.1016/j.neulet.2010.04.066 20447444

[B42] Sheinkopf S. J. Mundy P. Oller D. K. Steffens M. (2000). Vocal atypicalities of preverbal autistic children. *J. Autism Dev. Disord.* 30 345–354. 11039860 10.1023/a:1005531501155

[B43] Shriberg L. D. Paul R. McSweeny J. L. Klin A. Cohen D. J. Volkmar F. R. (2001). Speech and prosody characteristics of adolescents and adults with high-functioning autism and asperger syndrome. *J. Speech Lang. Hear. Res.* 44 1097–1115. 10.1044/1092-4388(2001/087) 11708530

[B44] Stylianou Y. (2001). Applying the harmonic plus noise model in concatenative speech synthesis. *IEEE Trans. Speech Audio Process.* 9 21–29. 10.1109/89.890068

[B45] Sun X. (2002). “Pitch determination and voice quality analysis using subharmonic-to-harmonic ratio,” in *Proceedings of the 2002 IEEE International Conference on Acoustics, Speech, and Signal Processing, Orlando, FL*, Vol. 1 (Piscataway, NJ: IEEE), 1–333.

[B46] Tabrikian J. Dubnov S. Dickalov Y. (2004). Maximum a-posteriori probability pitch tracking in noisy environments using harmonic model. *IEEE Trans. Speech Audio Process.* 12 76–87. 10.1109/tsa.2003.819950

[B47] Talkin D. Kleijn W. B. Paliwal K. K. (1995). “A robust algorithm for pitch tracking (RAPT),” in *Speech Coding and Synthesis*, eds Kleijn W. B. Paliwal K. K. (Amsterdam: Elsevier).

[B48] Tibshirani R. (1996). Regression shrinkage and selection via the lasso. *J. R. Stat. Soc. Ser. B* 58 267–288. 10.1111/j.2517-6161.1996.tb02080.x

[B49] Trevarthen C. Daniel S. (2005). Disorganized rhythm and synchrony: early signs of autism and rett syndrome. *Brain Dev.* 27 S25–S34. 16182487 10.1016/j.braindev.2005.03.016

[B50] Truong Q. T. Kato T. Yamamoto S. (2018). “Automatic assessment of L2 english word prosody using weighted distances of F0 and intensity contours,”in *Proceedings of the International Speech Communication Association.* 2186–2190.

[B51] van Santen J. P. Prud’hommeaux E. T. Black L. M. (2009). Automated assessment of prosody production. *Speech Commun.* 51 1082–1097. 10.1016/j.specom.2009.04.007 20160984 PMC2753987

[B52] Waibel A. (1988). *Prosody and Speech Recognition.* Burlington, MA: Morgan Kaufmann.

[B53] Wechsler D. (2003). *WISC-IV: Administration and Scoring Manual.* San Antonio, TX: Psychological Corporation.

[B54] Xu D. Richards J. A. Gilkerson J. (2014). Automated analysis of child phonetic production using naturalistic recordings. *J. Speech Lang. Hear. Res.* 57, 1638–1650. 10.1044/2014_JSLHR-S-13-0037 24824489

[B55] Yatawatte H. Poellabauer C. Latham S. (2016). “Automated capture of naturalistic child vocalizations for health research,” in *Proceedings of the 7th ACM International Conference on Bioinformatics, Computational Biology, and Health Informatics*, 472–473.

[B56] Zhang M. Xu S. Chen Y. Lin Y. Ding H. Zhang Y. (2021). Recognition of affective prosody in autism spectrum conditions: a systematic review and meta analysis. *Autism* 1362361321995725. 10.1177/1362361321995725 [Epub ahead of print]. 33722094

